# Editorial: Insights into the molecular dynamics of stress physiology in allium crops

**DOI:** 10.3389/fpls.2026.1825911

**Published:** 2026-03-24

**Authors:** Ofir Degani, Kiran Khandagale, Suresh Gawande

**Affiliations:** 1 Faculty of Sciences, Tel-Hai University of Kiryat Shmona and the Galilee, Upper Galilee, Tel-Hai, Israel; 2 Migal - Galilee Research Institute, Kiryat Shmona, Israel; 3 ICAR-Directorate of Onion and Garlic Research, Pune, India

**Keywords:** abiotic stress, allium crops, crop resilience, molecular mechanisms, stress physiology

Understanding stress physiology and molecular response mechanisms in plants is essential for improving crop production and quality, particularly under environmental stress. The genus *Allium* comprises some of the most economically and nutritionally important vegetable crops worldwide, including onion (*Allium cepa* L.), garlic (*Allium sativum* L.), and Welsh onion (*Allium fistulosum* L.) (Griffiths et al., 2002; Brewster, 2008). Despite their global cultivation, *Allium* crops are highly susceptible to a wide range of biotic (diseases, pests, and viruses) and abiotic (drought, salinity, waterlogging, temperature extremes, and nutrient deficiencies) stresses that affect growth, yield, storage quality, and market stability (Kale et al.). With climate change and global warming, intensifying salinity, erratic rainfall, flooding events, and pathogen pressure, understanding the molecular and physiological bases of stress resilience in *Allium* species has become increasingly important (Chaudhry et al., 2023). This Research Topic assembles five contributions that collectively advance our understanding of stress perception, metabolic regulation, defense mechanisms, disease ecology, and physiological disorders in *Allium* crops. Together, these studies illustrate that stress responses in *Allium* are multilayered, dynamic, and interconnected across molecular, physiological, and ecological scales (**Figure 1**). The findings are essential for cultivation programs and sustainable agriculture to improve crop production and quality.

**Figure 1:**
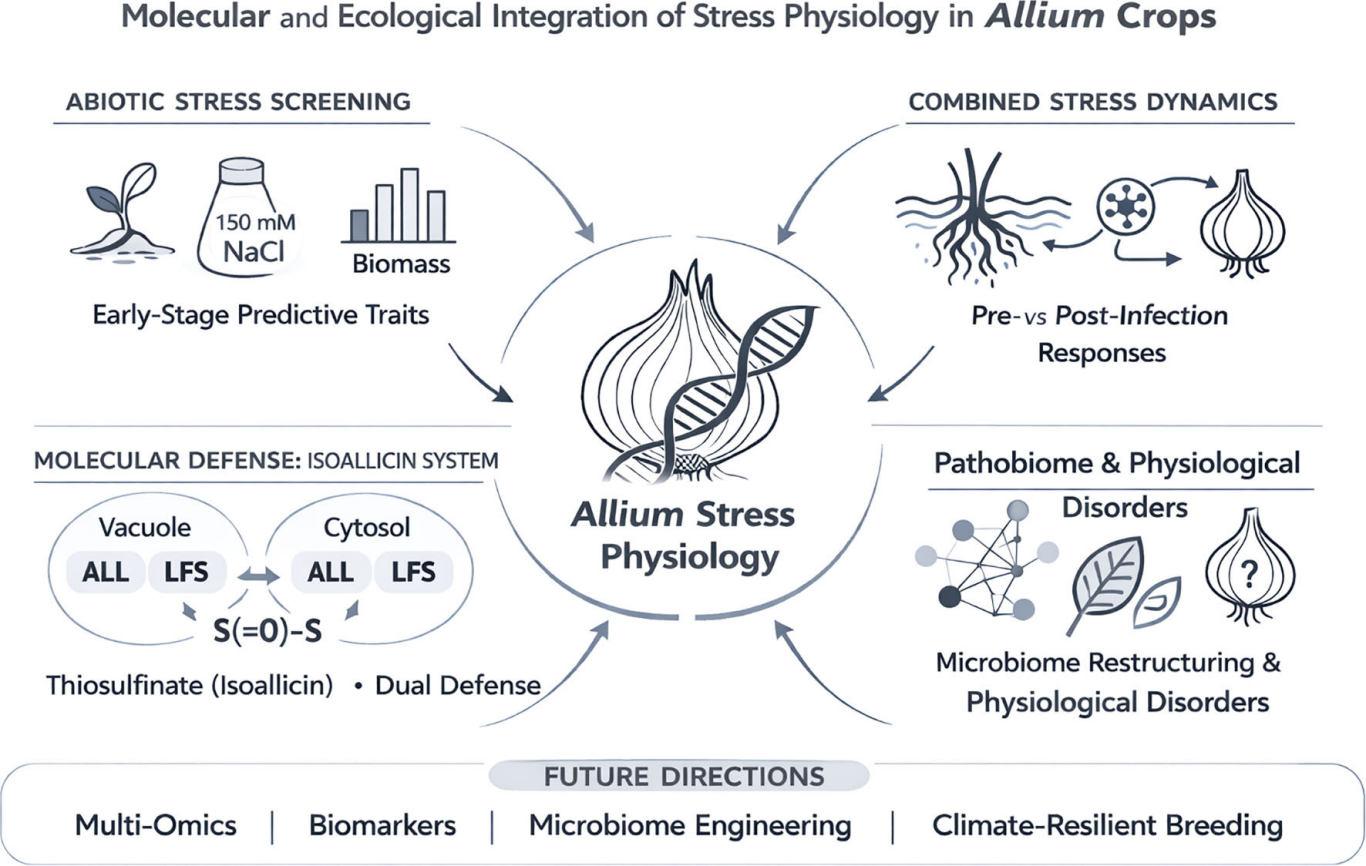
Molecular and ecological integration of stress physiology in *Allium* crops. Schematic overview of the multiscale mechanisms underlying stress responses in *Allium* species. The central node represents integrated *Allium* stress physiology, linking molecular, physiological, and ecological processes. Four interconnected domains are illustrated: (1) Abiotic stress screening, highlighting early-stage predictive traits for salinity tolerance and biomass-based selection approaches; (2) Combined stress dynamics, emphasizing the temporal effects of sequential abiotic and biotic stresses (e.g., pre- vs. post-infection waterlogging) on physiological outcomes; (3) Molecular defense architecture, depicting the dual isoallicin system involving cytosolic and vacuolar alliinases (ALL) and lachrymatory factor synthase (LFS), enabling both constitutive and inducible defense responses; and (4) Pathobiome and physiological disorders, illustrating microbiome restructuring and non-infectious abnormalities that affect yield and storage quality. The bottom panel outlines future research directions, including multi-omics integration, biomarker development, microbiome engineering, and climate-resilient breeding strategies. Arrows indicate functional interconnections among these domains, reflecting the dynamic and integrated nature of stress adaptation in *Allium* crops.

## Early-stage salinity screening and predictive traits

Salinity is one of the most severe abiotic constraints limiting onion productivity, particularly in salt-affected soils and irrigated systems. Kasar et al. conducted a large-scale screening of 116 onion genotypes at the germination stage to establish a reliable protocol for assessing salinity tolerance. By identifying 150 mM NaCl as an optimal discriminatory concentration and integrating morphological parameters with multivariate analyses, including principal component analysis and membership function modeling, the authors categorized genotypes into graded tolerance groups ranging from highly tolerant to highly sensitive. Importantly, total fresh weight under saline conditions emerged as a robust and practical screening trait. This work provides breeders with a reproducible early-stage selection framework and highlights that seedling-stage performance may serve as a predictive indicator of long-term field resilience. The study reinforces the importance of integrating quantitative modeling approaches into physiological screening for climate-resilient breeding.

## Temporal dynamics of combined abiotic and biotic stress

Climate change increasingly exposes crops to overlapping stress events, necessitating investigation beyond single-stress paradigms. Salunkhe et al. examined the interaction between waterlogging and anthracnose disease caused by *Colletotrichum gloeosporioides* species complex in onion. Their study revealed that stress timing critically shapes disease outcomes. Post-infection waterlogging significantly intensified disease severity, reduced chlorophyll and carotenoid content, decreased relative water content, elevated membrane injury, and triggered strong antioxidant responses (SOD and APX). In contrast, pre-infection waterlogging partially suppressed pathogen establishment, suggesting hypoxia-induced defense priming. These findings demonstrate that stress sequencing determines physiological exhaustion versus tolerance, emphasizing the importance of temporal context in stress physiology.

This work contributes to a growing body of evidence that cross-tolerance and stress priming are central mechanisms in plant adaptation. For *Allium* crops cultivated in monsoon climates or poorly drained soils, understanding such stress interactions is essential for both management strategies and breeding programs.

## Chemical defense architecture: the isoallicin system

A defining feature of *Allium* species is their sulfur-based defense chemistry. Cho et al. provided a comprehensive “isoallicin-omics” analysis of onion defense metabolism using genomic, transcriptomic, and metabolomic approaches. Mining the DHW30006 onion genome, the authors identified 64 alliinase (ALL) genes and 29 lachrymatory factor synthase (LFS) genes, key enzymes in isoallicin and lachrymatory factor biosynthesis. A particularly significant discovery was the presence of two functional categories of alliinases: vacuolar signal peptide-containing ALLs and cytosolic non-SP ALLs. This dual system suggests a layered defense strategy. Cytosolic alliinases maintain a basal production of isoallicin in intact tissues, functioning as a phytoanticipin defense, while vacuolar alliinases amplify isoallicin production upon tissue damage. Traditionally, isoallicin production was considered exclusively damage-induced. This study redefines the system as both constitutive and inducible, reflecting a sophisticated spatial and temporal regulation of defense metabolism. Such diversification of gene families and subcellular compartmentalization underscores the evolutionary adaptation of Allium crops to biotic stress.

## Microbiome restructuring and the pathobiome concept

Disease outcomes are increasingly understood within the context of the plant microbiome. Jayasinghe et al. investigated fungal community composition associated with leaf blight of Welsh onion in Taiwan using ITS1 amplicon sequencing. Their results revealed that leaf blight is a disease complex primarily involving *Stemphylium* and *Colletotrichum*, alongside shifts in phyllosphere and rhizosphere fungal communities. Symptomatic plants exhibited altered alpha-diversity patterns and restructured co-occurrence networks, supporting the “pathobiome” concept in which multiple taxa interact to drive disease progression. Rather than a single causal agent, disease emerges from community-level interactions shaped by environmental conditions and host physiology. This systems-level perspective opens new avenues for microbiome-based diagnostics and disease management strategies. It also aligns with broader ecological frameworks, recognizing that stress responses extend beyond host gene expression to encompass microbial assemblages.

## Physiological disorders: a hidden dimension of stress biology

Beyond abiotic and biotic stresses, physiological disorders represent a major yet underappreciated constraint on *Allium* production. Kale et al. provided the first comprehensive review and bibliometric assessment of physiological disorders in onion and garlic, including premature bolting, sprouting, doubles, watery scale, thick neck, and rubberization. Their analysis revealed increasing scientific attention over recent decades but persistent knowledge gaps in linking environmental triggers, genetic regulation, and management strategies. Physiological disorders can account for substantial yield and storage losses, yet they remain insufficiently integrated into molecular and breeding frameworks. By combining bibliometric visualization with critical assessment, the authors underscore the need to bridge agronomic practices, environmental modeling, and molecular physiology to mitigate these non-infectious abnormalities.

## Concluding remarks

Despite their economic and nutritional importance, the physiological and molecular mechanisms underlying stress responses in *Allium* crops remain underexplored compared to those in other major crop species. Across the contributions in this Research Topic, several converging themes and forward-looking directions emerge. Stress responses in *Allium* crops cannot be adequately interpreted within single-factor frameworks, as resilience is shaped by the temporal sequencing and interaction of abiotic and biotic stresses. Metabolic plasticity stands out as a central adaptive mechanism, with sulfur metabolism, antioxidant regulation, osmotic adjustment, and biomass allocation collectively underpinning stress tolerance. Early-stage phenotypes, particularly during germination and seedling development, provide valuable predictive indicators of subsequent field performance and represent practical targets for breeding programs. Disease development further reflects microbiome–host interdependence, in which shifts in community structure and microbial network dynamics significantly influence plant health outcomes. Despite substantial advances at the molecular and physiological levels, translational gaps remain, emphasizing the need to integrate mechanistic insights into breeding pipelines and field-scale management strategies to support climate-resilient *Allium* production systems. Future research should prioritize multi-omics integration under combined stress scenarios, the identification of robust predictive biomarkers, functional validation of defense-related gene families, and microbiome engineering approaches. Moreover, incorporating research on physiological disorders into climate-resilience frameworks will be essential for safeguarding yield stability and postharvest quality.
